# Theoretical investigation and practical validation of AI based fault detection in BJT amplifier circuits

**DOI:** 10.1038/s41598-025-28148-6

**Published:** 2025-12-03

**Authors:** Kyrillos K. Selim, Ahmed Alaa, Ola Hassan, Sameh O. Abdellatif

**Affiliations:** 1 Department of Electronics Technology, Faculty of Technology and Education, Helwan University, Cairo, 11795 Egypt; 2 The Electrical Engineering Department and FabLab at The Centre of Emerging Learning Technologies, CELT, British University in Egypt (BUE), Cairo, 11387 Egypt

**Keywords:** Analog circuits, BJT circuits, Fault detection, Machine learning, Feature extraction, Random forest, Engineering, Mathematics and computing

## Abstract

Analog circuits are essential to electronic systems due to their variety of critical functions. Catastrophic faults, such as short- and open-circuit faults, can stop the circuit from functioning. Meanwhile, parametric faults such as drift due to temperature fluctuations, noise in components, and power supply issues cause the circuit to operate beyond its range. Such faults can harm the circuit and its constituent parts. Therefore, it is crucial to identify and address the issues brought on by these faults. Common traditional fault diagnosis methods that rely on manual testing and visual assessment are lacking in complex electronic systems and faults. Such methods can catch obvious problems but struggle with subtle or rare faults. Herein, AI has emerged as a strong tool in enhancing fault detection processes, providing capabilities for analyzing vast amounts of data, recognizing patterns, and making decisions based on that data with improved accuracy and speed in fault diagnosis. Accordingly, the common emitter circuit—DC configuration was considered with 15 patterns. The first pattern represents normal conditions, whereas the others represent faulty conditions. The faulty patterns included probable fluctuations in collector and emitter resistor values, power supply internal resistance, and operating temperature. A dataset covering the study parameters and circuit patterns was established, considering both simulation and experimental readings. Supervised machine learning, primarily using random forest classifiers, was employed to detect faults. The hybrid dataset model was trained and validated on that basis. The system achieved a 98.7% success rate in the fault detection condition, demonstrating its efficiency and robust performance. To further investigate the physics behind various fault patterns, a comprehensive analytical model was developed and linked to the machine learning results, highlighting the effectiveness of each feature.

## Introduction

Analog circuits have suffered significantly from varied faults hindering their proper operation^1^. The two primary classifications for these faults are parametric and catastrophic^2^. When catastrophic faults occur, the circuit completely stops functioning^3^. Such faults involve ground faults, in which a signal or component is shorted to ground; short-circuit faults, characterized by unintended electrical contact between component leads or conductive pathways; and open circuit faults, in which a component acts as if it were disconnected^4^. These faults could cause the circuit to malfunction^4^. On the other hand, parametric faults can lead to inaccurate outcomes or the circuit operating beyond the proper range^5^. Such faults include drift due to temperature fluctuations, noise in components caused by signal disturbances, and power supply issues like overvoltage or under-voltage situations^2,6–9^. Catastrophic and parametric faults both cause harm to the circuit and its constituent parts^10^. Therefore, to ensure that the circuit functions as intended, it is crucial to identify and address the issues brought on by these faults^6^.

As highlighted earlier, fault detection in electronic circuits plays a significant role in modern electrical systems for safety and reliability reasons^11^. Prompt detection and diagnosis may prevent damage to the equipment, reduce downtime, and uphold the efficiency of the operation. Conventional methods for fault detection may involve manual checks and routine preprogrammed testing procedures, which are very time-consuming and possibly unable to pinpoint all the prospective problems^12^. Traditional approaches are lacking in the case of complex electronic systems and faults^12^. Traditional methods depend on manual testing and visual checks, where these approaches can catch obvious problems but struggle with subtle or rare faults. As circuits get more complex, manual testing becomes slower and less reliable^6^. Recently, it has emerged as a strong tool in enhancing fault detection processes: artificial intelligence (AI)^13–16^. AI techniques, such as machine learning^17,18^ and deep learning^19^, offer powerful tools for processing large-scale datasets, identifying complex patterns, and enabling data-driven decision-making. Such techniques, therefore, provide enhanced accuracy and speed in fault diagnosis^20,21^. AI-based models have also been applied to electrical machines to detect faults with much better performance than traditional methods^16,22^. Hence, AI-based techniques overcome methodological limitations and challenges. It processes large amounts of data rapidly and spots patterns beyond human perception. Machine learning models analyze various measurements, such as voltage, current, and gain, to find irregularities. Additionally, techniques like neural networks and decision trees can adapt to new fault types without requiring major updates^6^. AI-based techniques also help with predictive maintenance^23^. It identifies early failure indicators, allowing fixes to be made before systems fail. This increases component life, reduces repair costs, and decreases downtime^23^. As electronics become more advanced, AI-based fault detection is no longer optional. It provides a faster, more accurate, and scalable way to keep systems running smoothly and efficiently^1,24^.

In the literature, Shokrolahi et al. proposed a method based on a deep neural network for fault detection in analog circuits, utilizing the spectrogram of signals as the image input to a convolutional neural network (CNN) to capture microstructural features across all classes^25^. After that, the deep neural network extracted discriminative features, and finally, classification was performed by using a simple SoftMax layer. The performance of the proposed simulation method was tested in two analog filters (Sallen-Key band-pass filter and band-stop filter), whereas the data were divided into two groups of training data and testing data so that 70% of the data (90 samples) were used randomly for network training and 30% (60 samples) were used for network testing^26^. In a similar direction, Gao et al. presented a fault diagnosis method based on conditional variational neural networks (CVNN). The method was fully evaluated with the three analog filters (Sallen–Key bandpass filter, four-Op-Amp biquad high-pass filter, and CTSV filter)^27^. The circuits were employed to conduct both simulations and experiments for fault diagnosis, and four fault degrees of analog circuits are utilized in these experiments according to the deviation of the fault component from the nominal value^27^. Zhao et al. proposed an analog circuit fault diagnosis method based on a deep belief network (DBN)^28,29^. Experiments on common soft faults and incipient faults of two test filter circuits (Sallen-Key bandpass filter and four-Op-Amp biquad high-pass filter) with varying complexities were presented to demonstrate the effectiveness of the proposed method. However, the authors mentioned that the drawback of their proposed method was that the features extracted by DBN have no physical meanings; later with the same adopted two filter circuits, Zhao used the generalized multiple kernel learning support vector machine (GMKL-SVM), to identify the incipient fault classes of analog circuits and adopted the particle swarm intelligent optimization algorithm, the sine cosine algorithm (SCA), to optimize key parameters of GMKL-SVM^28,29^.

AI approaches for fault detection and classification have been the focus of several research investigations. Literature has extensively examined the use of machine learning algorithms^30–32^. Various methods for classifying faults in analog circuits using neural network classifiers were presented. A unique version of conventional and conditional variational neural network techniques was introduced in^27,33^, and one of them was based on multifrequency measurements and an estimate of the component faulty value^34^. By employing diverse techniques, deep learning algorithms have become popular for classifying analog faults^26,28,35–37^. Additional techniques included the use of genetic algorithms^38^ and support vector machine algorithms^39^. Considering the relevant literature on fault detection in electronic circuits, it is evident that the various AI techniques adopted for this purpose, as previously mentioned, have been primarily evaluated using op-amp-based filter circuits, as seen in^6,40–46^.

Moreover, the unavailability of bipolar junction transistor (BJT) fault detection is adopted in the existing literature. Some of the main difficulties are the general lack of similar studies, specifically on fault detection in BJT circuits using AI, which introduces significant challenges in the process of finding either reference methodologies or benchmark datasets. Moreover, challenges in data collection and labeling: Generally speaking, any AI model training requires huge amounts of datasets with labeled fault and non-fault conditions. For BJT circuits, this could be a painstaking process when trying to acquire real-world data. Furthermore, various AI methods, such as the Support Vector Machine, Quadratic Discriminant Analysis, and Extreme Learning Machine, give different performances in terms of accuracy and computation time. Hence, a wide range of tests and performance measurements is necessary for arriving at the optimum model for BJT fault detection.

This work intends to investigate the application of AI techniques in fault detection within BJT circuits. This study aims to apply AI to develop a fault detection system that is more efficient and accurate in addressing the complexities inherent in modern electronic circuits. Additionally, this paper carries out extensive simulations and experiments to generate original data and validate my approach. The unavailability of datasets will be minimized by using synthetic fault datasets generated through circuit simulation software, combined with experimentally measured values. Finally, various AI models were implemented and then compared using measurements such as performance, accuracy, precision, recall, or computation speed to find which one best suited the proposed application. To link the AI outputs with theoretical understanding, an analytical model is provided. The subsequent sections outline the proposed “analytical results” and the AI-based approach and present the results of implementation in BJT circuits in the “Methodology” and “Results and Discussion” sections. Finally, the “Conclusion” section briefly presents the proposed method and the most important findings achieved by this study, and potential directions for future work in this research field.

## Theoretical investigation

**Fig. 1:**
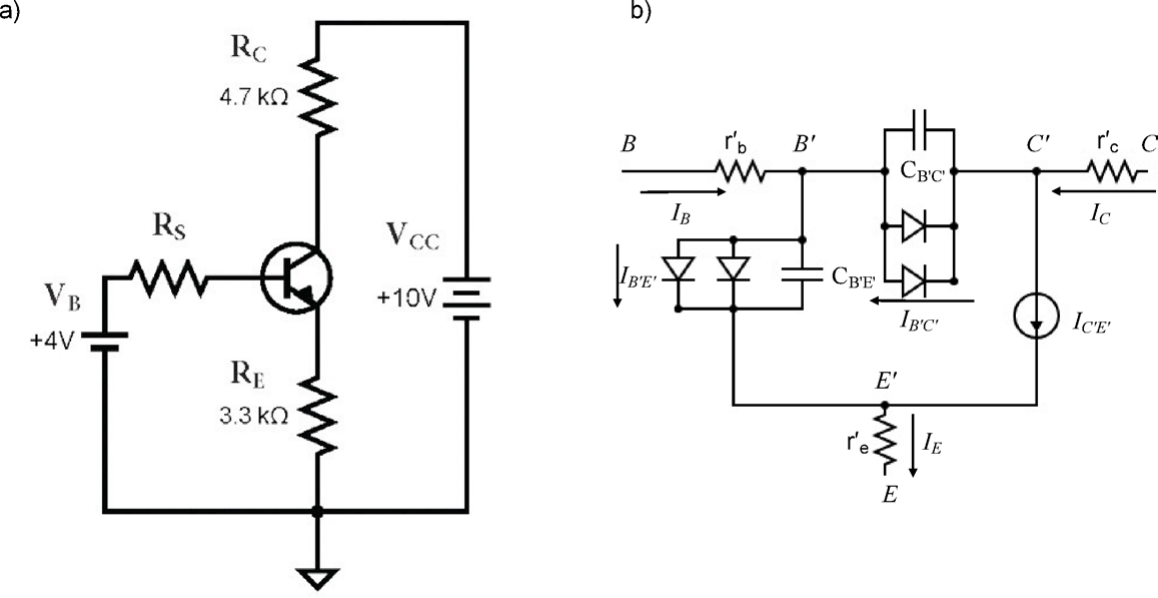
(**a**) The common emitter BJT-based circuit, (**b**) BJT small-circuit model.

As indicated earlier in the introduction, the focus of this study is to investigate the utilization of machine learning approaches in exploring various faults in a basic BJT-based amplifier circuit, see Fig. 1-a. In this context, we explore faults related to variation in the operating temperature, as well as the tolerance in the resistances associated with the three terminals of the transistors. For such a purpose, introduce an analytical approach to deeply understand the physics beyond both resistive and thermal variation, highlighting the expected impact on the circuit operating conditions. To address the analytical approach, we adopted the small circuit model in Fig. 1-b, while following the approach in^47^. Herein, *B’*, *E’*, and *C’* indicate the intrinsic base, emitter, and collector terminals of the BJT without considering the parasitic resistance effect, respectively, while *B*, *E*, and *C* indicate intrinsic base, emitter, and collector terminals of the BJT considering the parasitic resistance effect. The following currents *I*_*B’C’*_ and *I*_*B’E’*_ indicate the intrinsic base-collector and base-emitter currents, respectively, flowing through their corresponding diode structures. *I*_*C’E’*_ indicates the intrinsic collector-emitter current, modeled as an ideal current source. Alternatively, the space-charge and diffusion capacitances are associated with the base-collector and base-emitter junctions, respectively, while r′_b_, r′_c_ and r′_e_ indicate the base, collector, and emitter ohmic parasitic elements, respectively. *I*_*B*_, *I*_*C*,_ and *I*_*E*_ indicate the base, collector and emitter current through their respective ohmic parasitic. The base-emitter current ($\:{I}_{B{\prime\:}E{\prime\:}}$) and the base-collector current ($\:{I}_{B{\prime\:}C{\prime\:}}$) can be added to generate the base current *I*_*B*_, as follows:

1 $$\:{I}_{B}=\:{I}_{B{\prime\:}E{\prime\:}}+\:{I}_{B{\prime\:}C{\prime\:}}$$

where $\:{I}_{B{\prime\:}E{\prime\:}}$, and $\:{I}_{B{\prime\:}C{\prime\:}}$ can be formulated as the summation of the ideal and a non-ideal component:

2-a $$\:{I}_{B{\prime\:}E{\prime\:}}=\frac{{I}_{F}}{{\beta\:}_{F}}+\:{I}_{BErec}$$

2-b $$\:{I}_{B{\prime\:}C{\prime\:}}=-(\frac{{I}_{R}}{{\beta\:}_{R}}+\:{I}_{BCrec})$$

since *I*_*F*_ is the ideal forward diffusion current, $\:\beta\:$_*F*_ is the ideal forward maximum current gain and *I*_*BErec*_ is the recombination current in the base-emitter junction. Additionally, *I*_*R*_, $\:{\beta\:}_{R}$, and $\:{I}_{BCrec}$ are defined as the ideal reverse diffusion current, ideal maximum reverse current gain, and recombination current through the base-collector junction, respectively, while considering active mode operation. The ideal forward and reverse diffusion currents are defined as:

3-a $$\:{I}_{F}=\:{I}_{S}\left(\text{exp}\left(\frac{{V}_{BE}}{{N}_{F}{\:V}_{th}}\right)-\:1\right)$$

3-b $$\:{I}_{R}=\:{I}_{S}\left(\text{exp}\left(\frac{{V}_{BC}}{{N}_{R}{\:V}_{th}}\right)-\:1\right)$$

where $\:{I}_{S}$ is the saturation current, $\:{\:V}_{th}$ is the thermal voltage, and $\:{N}_{F}$ and $\:{N}_{R}$ correspond to the forward and reverse current emission coefficients, respectively. The recombination currents at the base-collector and base-emitter junctions are expressed as follows:

4-a $$\:{I}_{BErec}=\:{I}_{SE}\left(\text{exp}\left(\frac{{V}_{BE}}{{N}_{E}{\:V}_{th}}\right)-\:1\right)$$

4-b $$\:{I}_{BCrec}=\:{I}_{SC}\left(\text{exp}\left(\frac{{V}_{BC}}{{N}_{C}\:{\:V}_{th}}\right)-\:1\right)$$

where $\:{I}_{SE}$ and $\:{I}_{SC}$ represent the leakage currents of the emitter and collector, respectively, while $\:{N}_{E}$ and $\:{N}_{C}$ denote their corresponding current emission coefficients. Accordingly, the base current can be expressed as:

$$\:{I}_{B=}\left(\frac{{I}_{S}}{{\beta\:}_{F}}\right)\times\:\:\left(\text{exp}\left(\frac{{V}_{BE}}{{N}_{F}\:\times\:\:{V}_{th}}\right)-\:1\right)+\:{I}_{SE}\:\times\:\:\left(\text{exp}\left(\frac{{V}_{BE}}{{N}_{E}\:\times\:\:{V}_{th}}\right)-\:1\right)$$

5 $$\:\:\:\:\:+\:\left(\frac{{I}_{S}}{{\beta\:}_{R}}\right)\times\:\:\left(\text{exp}\left(\frac{{V}_{BC}}{{N}_{R}\:\times\:\:{V}_{th}}\right)-\:1\right)+\:{I}_{SC}\:\times\:\:\left(\text{exp}\left(\frac{{V}_{BC}}{{N}_{C}\times\:\:{V}_{th}}\right)-\:1\right)$$

The current of the collector ($\:{I}_{C}$) can be formulated by the addition of the collector-emitter current and the base-collector current, as expressed below:

6 $$\:{I}_{C}=\:{I}_{C{\prime\:}E{\prime\:}}+{I}_{B{\prime\:}C{\prime\:}}=\:\frac{{I}_{F}\:-\:{I}_{R}}{{N}_{qb}}+\:\frac{{I}_{R}}{{\beta\:}_{R}}+\:{I}_{BCrec}$$

where $\:{N}_{qb}$ represents the base charge expression, accounting for device non-idealities such as base-width modulation and high-level injection effects. It is defined as the normalized base charge under a given bias condition relative to its value in the unbiased state. This concept is formulated as below:

7 $$\:{\text{N}}_{\text{q}\text{b}}\:=\:\frac{{\text{q}}_{1\text{s}}}{2}\:\left(1\:+\:\sqrt{1\:+\:4{\text{q}}_{2\text{s}}}\right)$$

The parameter $\:{\text{q}}_{1\text{s}}$ captures the effect of base-width modulation, while $\:{\text{q}}_{2\text{s}}$ accounts for the high-level injection phenomenon. These effects are modeled as the following:

8-a $$\:{q}_{1s}=\frac{1}{1-\frac{{V}_{BE}}{{V}_{AR}}-\frac{{V}_{BC}}{{V}_{AF}}}$$

8-b $$\:{q}_{2s}=\frac{{I}_{S}}{{I}_{KF}}\left(\text{exp}\left(\frac{{V}_{BE}}{{N}_{F}\cdot\:{V}_{th}}\right)-1\right)+\frac{{I}_{S}}{{I}_{KR}}\left(\text{exp}\left(\frac{{V}_{BC}}{{N}_{R}\cdot\:{V}_{th}}\right)-1\right)$$

where $\:{V}_{AF}$, $\:{V}_{AR}$, $\:{I}_{KF}$ and $\:{I}_{KR}\:$are the forward and reverse early voltages, and forward and reverse knee currents, respectively. Hence, the current of the emitter can be calculated by the summation of $\:{I}_{B}\:and\:{I}_{C}$:

9 $$\:{I}_{E}=\:{I}_{B}+\:{I}_{C}$$

The equations describing the relationship between the intrinsic junction voltages $\:{V}_{\text{B'E'}}$and $\:{V}_{\text{B'C'}}$ and their corresponding terminal voltages $\:{V}_{\text{BE}}$ and $\:{V}_{\text{BC}}$ are defined as the following:

10-a $$\:{V}_{\text{B'E'}}={V}_{\text{BE}}-\left({I}_{B}{r}_{\text{BB}}\left({I}_{B}\right)+{I}_{E}{{r}^{{\prime\:}}}_{E}\right)$$

10-b $$\:{V}_{\text{B'C'}}={V}_{\text{BC}}-\left({I}_{B}{r}_{\text{BB}}\left({I}_{B}\right)-{I}_{C}{{r}^{{\prime\:}}}_{C}\right)$$

The base resistance $\:{r}_{BB}\left({I}_{B}\right)\:$according to the SPICE Gummel-Poon (SGP) model is formulated as the following^47^:

11-a $$\:{r}_{BB}\left({I}_{B}\right)={r}_{BM}+3\left({r{\prime\:}}_{B}-{r}_{BM}\left[\frac{\text{tan}z-z}{z{\text{tan}}^{2}z}\right]\right)$$

11-b $$\:z=\frac{\sqrt{1+{\left(\frac{12}{{\uppi\:}}\right)}^{2}\frac{{I}_{B}}{{I}_{RB}}}-1}{\frac{24}{{{\uppi\:}}^{2}}\sqrt{\frac{{I}_{B}}{{I}_{RB}}}}$$

where $\:{r{\prime\:}}_{B}$ denotes the zero-bias base resistance, while $\:{r}_{BM}$ represents the minimum base resistance observed at high base current levels. $\:{I}_{RB}$ corresponds to the base current at which the base resistance$\:\:{r}_{BB}\left({I}_{B}\right)$ decreases to the average value between $\:{r{\prime\:}}_{B}$ and $\:{r}_{BM}$. The depletion capacitances of the base-emitter (BE) and base-collector (BC) junctions are defined as the sum of their respective space-charge and diffusion capacitance components, as below:

12-a $$\:{C}_{B{\prime\:}E{\prime\:}}=\frac{{C}_{JE}}{{\left(1-\frac{{V}_{BE}}{{V}_{\text{B'E'}}}\right)}^{{M}_{JE}}}$$

12-b $$\:{C}_{B{\prime\:}C{\prime\:}}=\frac{{C}_{JC}}{{\left(1-\frac{{V}_{BC}}{{V}_{\text{B'C'}}}\right)}^{{M}_{JC}}}$$

where $\:{C}_{JE}$and $\:{C}_{JC}$ represent the zero-bias capacitances of the base-emitter and base-collector junctions, respectively, while $\:{M}_{JE}$ and $\:{M}_{JC}$ denote the corresponding junction grading (exponential) coefficients. The temperature-dependent auxiliary variables in the SGP model are defined as below:

13-a $$\:{V}_{th}=\frac{kT}{q}$$

13-b $$\:{E}_{G}=1.16-\frac{7.02\times\:{10}^{-4}{T}^{2}}{T+1108}$$

13-c $$\:{n}_{i}=1.45\times\:{10}^{10}{\left(\frac{T}{{T}_{0}}\right)}^{1.5}\text{exp}\left[\frac{q}{2k}\left(-\frac{{E}_{G}}{T}+\frac{1.1151}{T}\right)\right]$$

where $\:{E}_{G}$, $\:{n}_{i}$, *q*, *k*, $\:T$, and $\:{T}_{0}\:$are the bandgap, intrinsic carrier concentration, electron charge, Boltzmann constant, junction temperature, and reference temperature (25 °C). The temperature-dependent parameters utilized in the SGP model are sequenced as follows:

14-a $$\:{I}_{S}\left(T\right)={I}_{S}\left({T}_{0}\right){\left(\frac{T}{{T}_{0}}\right)}^{XTI}(\text{exp}\left[\frac{{qE}_{G}}{{V}_{th}}\left(\frac{T}{{T}_{0}}\right)\right]-1)$$

14-b $$\:{\beta\:}_{F}\left(T\right)={\beta\:}_{F}\left({T}_{0}\right){\left(\frac{T}{{T}_{0}}\right)}^{XTB}$$

14-c $$\:{\beta\:}_{R}\left(T\right)={\beta\:}_{R}\left({T}_{0}\right){\left(\frac{T}{{T}_{0}}\right)}^{XTB}$$

where $\:{\beta\:}_{F}\left({T}_{0}\right)$ is the forward common-emitter current gain at the standard reference temperature $\:(25^\circ\:\text{C})$, which is given by 134.4 in the current study; $\:{\beta\:}_{R}\left({T}_{0}\right)$ is the reverse common-emitter current gain at the standard reference temperature $\:(25^\circ\:\text{C})$, which is $\:0.95$^48^$\:.\:$Moreover, $\:XTI$ and $\:XTB$ are the temperature exponents for the effect of $\:{I}_{S}$, forward-, and reverse-beta temperature exponent^49^.

15-a $$\:{I}_{SE}\left(T\right)={I}_{SE}\left({T}_{0}\right){\left(\frac{T}{{T}_{0}}\right)}^{-XTB}\frac{{I}_{S}{\left(T\right)}^{\frac{1}{{N}_{E}}}}{{I}_{S}\left({T}_{0}\right)}$$

15-b $$\:{I}_{SC}\left(T\right)={I}_{SC}\left({T}_{0}\right){\left(\frac{T}{{T}_{0}}\right)}^{-XTB}\frac{{I}_{S}{\left(T\right)}^{\frac{1}{{N}_{C}}}}{{I}_{S}\left({T}_{0}\right)}$$

15-c $$\:{V}_{JE}\left(T\right)={V}_{JE}\left({T}_{0}\right)\left(\frac{T}{{T}_{0}}\right)+2{V}_{th}\text{log}\left[\frac{1.45\times\:{10}^{10}}{{n}_{i}}\right]$$

15-d $$\:{V}_{JC}\left(T\right)={V}_{JC}\left({T}_{0}\right)\left(\frac{T}{{T}_{0}}\right)+2{V}_{th}\text{log}\left[\frac{1.45\times\:{10}^{10}}{{n}_{i}}\right]$$

In the current work, the target is to monitor the fault variations due to operating temperature, as well as resistances, that impact the transistor terminal currents or voltages. Herein, the derivation above is customized to show a set of governing equations that facilitate our physical understanding of the impact of these possible fault scenarios on the transistor terminals’ currents and voltages. The following set of equations is used to describe the five main observing parameters in this study:

$$\:{I}_{B}\left(T\right)=\left(\frac{{I}_{S}\left(T\right)}{{\beta\:}_{F}\left(T\right)}\right)\times\:\:\left(\text{exp}\left(\frac{{V}_{BE}\left(T\right)}{{N}_{F}\:\times\:\:{V}_{th}\left(T\right)}\right)-\:1\right)$$

$$+\:{I}_{SE}\left(\text{T}\right)\:\times\:\:\left(\text{exp}\left(\frac{{V}_{BE}\left(T\right)}{{N}_{E}\:\times\:\:{V}_{th}\left(T\right)}\right)-\:1\right)$$

16-a $$\:\:\:\:\:+\:\left(\frac{{I}_{S}\left(T\right)}{{\beta\:}_{R}\left(T\right)}\right)\times\:\:\left(\text{exp}\left(\frac{{\varvec{V}}_{\varvec{B}\varvec{C}}\:\left(\varvec{T}\right)}{{N}_{R}\:\times\:\:{V}_{th}\left(T\right)}\right)-\:1\right)+\:{I}_{SC}\left(\text{T}\right)\:\times\:\:\left(\text{exp}\left(\frac{{\varvec{V}}_{\varvec{B}\varvec{C}}\left(\varvec{T}\right)}{{N}_{C}\times\:\:{V}_{th}\left(T\right)}\right)-\:1\right)$$

$$\:{I}_{C}\left(T\right)\:=\:\frac{{I}_{S}\left(T\right)\left(\text{exp}\left(\frac{{\varvec{V}}_{\varvec{B}\varvec{E}}\left(\varvec{T}\right)}{{N}_{F}{\:V}_{th}\left(T\right)}\right)\:-\:1\right)\:-\:{I}_{S}\left(T\right)\left(\text{exp}\left(\frac{{\varvec{V}}_{\varvec{B}\varvec{C}}\left(\varvec{T}\right)}{{N}_{R}{\:V}_{th}\left(T\right)}\right)\:-\:1\right)}{{N}_{qb}}$$

16-b $$\:-\:\frac{{I}_{S}\left(T\right)\left(\text{exp}\left(\frac{{\varvec{V}}_{\varvec{B}\varvec{C}}\left(\varvec{T}\right)}{{N}_{R}{\:V}_{th}\left(T\right)}\right)\:\:-\:1\right)}{{\beta\:}_{R}\left(T\right)}-\:{I}_{BCrec}\:\left(T\right)$$

16-c $$\:{I}_{E}\left(\text{T}\right)=\:{I}_{B}\left(\text{T}\right)+\:{I}_{C}\left(T\right)$$

16-d $$\:{V}_{\text{BE}}\left(\text{T}\right)={V}_{\text{B'E'}}+\left({I}_{B}\left(T\right)\:{(r}_{\text{BB}}\left(\text{T}\right)+{R}_{S}\left)\right)+{I}_{E}{{\left(T\right)\:(r}^{{\prime\:}}}_{E}+{R}_{E}\right)$$

16-e $$\:{V}_{\text{BC}}\left(\text{T}\right)={V}_{\text{B'C'}}+\:\left({I}_{B}\left(T\right)\:{(r}_{\text{BB}}\left(\text{T}\right)+{R}_{S}\left)\right)-{I}_{C}\left(T\right)\:{{(r}^{{\prime\:}}}_{C}+{R}_{C}\right)$$

Accordingly, a set of associated equations is formulated as:

17-a $$\:{I}_{S}\left(T\right)={I}_{S}\left({T}_{0}\right){\left(\frac{T}{{T}_{0}}\right)}^{XTI}\text{exp}\left[\frac{{qE}_{G}}{{V}_{th}\left(T\right)}\left(\left(\frac{T}{{T}_{0}}\right)-1\right)\right]$$

17-b $$\:XTI=\left(\text{exp}\left(\frac{{\varvec{V}}_{\varvec{B}\varvec{E}}\left(T\right)}{{N}_{F}{\:V}_{th}\left(T\right)}\right)-\:1\right)$$

17-c $$\:{V}_{th}\left(\text{T}\right)=\frac{kT}{q}$$

17-d $$\:{\beta\:}_{F}\left(T\right)={\beta\:}_{F}\left({T}_{0}\right){\left(\frac{T}{{T}_{0}}\right)}^{XTB}$$

17-e $$\:XTB=\:\left(\text{exp}\left(\frac{{\varvec{V}}_{\varvec{B}\varvec{C}}\left(T\right)}{{N}_{R}{\:V}_{th}\left(T\right)}\right)\:-\:1\right)$$

17-f $$\:{I}_{SE}\left(T\right)={I}_{SE}\left({T}_{0}\right){\left(\frac{T}{{T}_{0}}\right)}^{-XTB}\frac{{I}_{S}{\left(T\right)}^{\frac{1}{{N}_{E}}}}{{I}_{S}\left({T}_{0}\right)}$$

17-g $$\:{I}_{SC}\left(T\right)={I}_{SC}\left({T}_{0}\right){\left(\frac{T}{{T}_{0}}\right)}^{-XTB}\frac{{I}_{S}{\left(T\right)}^{\frac{1}{{N}_{C}}}}{{I}_{S}\left({T}_{0}\right)}$$

17-h $$\:{\beta\:}_{R}\left(T\right)={\beta\:}_{R}\left({T}_{0}\right){\left(\frac{T}{{T}_{0}}\right)}^{XTB}$$

17-i $$\:{\text{N}}_{\text{q}\text{b}}\:=\:\frac{{\text{q}}_{1\text{s}}}{2}\:\left(1\:+\:\sqrt{1\:+\:4{\text{q}}_{2\text{s}}}\right)$$

17-j $$\:{q}_{1s}=\frac{1}{1-\frac{{V}_{BE\left(T\right)}}{{V}_{AR}}-\frac{{V}_{BC}\left(T\right)}{{V}_{AF}}}$$

17-k $$\:{q}_{2s}=\frac{{I}_{S}\left(T\right)}{{I}_{KF}}\left(\text{exp}\left(\frac{{V}_{BE}\left(T\right)}{{N}_{F}\cdot\:{V}_{th}\left(T\right)}\right)-1\right)+\frac{{I}_{S}\left(T\right)}{{I}_{KR}}\left(\text{exp}\left(\frac{{V}_{BC}\left(T\right)}{{N}_{R}\cdot\:{V}_{th}\left(T\right)}\right)-1\right)$$

17-l $$\:{I}_{BCrec}=\:{I}_{SC}\left(T\right)\left(\text{exp}\left(\frac{{V}_{BC}\left(T\right)}{{N}_{C}\:{\:V}_{th}\left(T\right)}\right)-\:1\right)$$

17-m $$\:{r}_{BB}\left({I}_{B}\right)={r}_{BM}+3\left({r{\prime\:}}_{B}-{r}_{BM}\left[\frac{\text{tan}z-z}{z{\text{tan}}^{2}z}\right]\right)$$

17-n $$\:z=\frac{\sqrt{1+{\left(\frac{12}{{\uppi\:}}\right)}^{2}\frac{{I}_{B}}{{I}_{RB}}}-1}{\frac{24}{{{\uppi\:}}^{2}}\sqrt{\frac{{I}_{B}}{{I}_{RB}}}}$$

Finally, Table 1 lists the values for the initial parameters seeded into the model. The demonstrated model is solved to extract the five parameters as a function of operating temperature and resistances, where results are utilized to provide a technical depth for the AI extracted results, as displayed in the results and discussion section. It is worth highlighting that the impact of the parasitic capacitance was ignored in this study, as the study is mainly focusing on the DC analysis of the amplifier BJT-based circuit.

**Table 1:** List of parameter values and constants seeded into the analytical model.

Parameter	Value/range	Unit
$\:{T}_{0}$	25	$\:{}^{o}C$
$\:{I}_{S}\left({T}_{0}\right)$	0.01	pA
$\:T$	20–100	$\:{}^{o}C$
$\:{E}_{G}$	1.12	eV
$\:{N}_{F}$	0.98	1
$\:k$	8.617 × 10⁻⁵	eV/K
$\:q$	1.6 × 10⁻^19^	C
$\:{\beta\:}_{F}\left({T}_{0}\right)$	134.4	1
$\:{N}_{R}$	150	1
$\:{I}_{SE}\left({T}_{0}\right)$	0.25	µA
$\:{N}_{E}$	0.98	1
$\:{\beta\:}_{R}\left({T}_{0}\right)$	0.95	1
$\:{N}_{C}$	200	1
$\:{I}_{SC}\left({T}_{0}\right)$	0.76	µA
$\:{V}_{AR}$	12	V
$\:{V}_{AF}$	75	V
$\:{I}_{KF}$	5	mA
$\:{I}_{KR}$	35	µA
$\:{r}_{BM}$	950	Ω
$\:{r{\prime\:}}_{B}$	100	Ω
$\:{R}_{S}$	0–1000	Ω
$\:{{r}^{{\prime\:}}}_{E}$	0.1	Ω
$\:{R}_{E}$	3.3	kΩ
$\:{{r}^{{\prime\:}}}_{C}$	70	Ω
$\:{R}_{C}$	4.7	kΩ

## Dataset generation

Following the aim of the current study to explore the utilization of machine learning algorithms in fault detection of BJT-based circuits, generating a sufficient dataset is a key ste Herein, we used a combination of simulated data and experimentally measured records to construct our dataset. The current study included four different fault patterns in addition to the normal pattern. These included the effects of high operating temperature, as well as unexpected variations in the extrinsic emitter and collector. Moreover, the effect of the DC source resistance, biasing the base terminal, is addressed and treated as an extrinsic base resistance. In the next section, the classification procedure is investigated. The Multisim Simulator was used in this study to simulate the common emitter circuit, as depicted in Fig. 1-a. The relevant simulation results were extracted to build the dataset. Regarding the fault detection for the common-emitter circuit considered, Table 2 introduces the different patterns for the current study, which includes both normal and faulty patterns.

In the normal configuration, the DC supply connected to the base is set to 4 V, while $\:{V}_{CC}$ is 10 V. The collector resistance is 4.7 kΩ, and the emitter resistance is 3.3 kΩ. The ideal values for the designed common-emitter (CE) circuit are labeled as “0” in the dataset, and the simulation results under these conditions align with the theoretical predictions. In the faulty configuration, we have four classifications, detailed in Table 2. The first classification includes four patterns, each with four variable sweeping parameters that represent fault sources potentially leading to inaccurate outcomes or causing the circuit to operate outside its intended range, negatively impacting performance. The first parameter is the temperature fluctuations ($\:T$), which were varied from 25 °C to 100 °C in increments of 5 °C, resulting in a total of 16 points. The second parameter is the collector resistor ($\:{R}_{C}$), which was adjusted from 4.5 kΩ to 4.8 kΩ in increments of 10 Ω, yielding a total of 31 points. The third parameter is the emitter resistor ($\:{R}_{E}$), swept from 3.1 kΩ to 3.4 kΩ, also in increments of 10 Ω, resulting in 31 points. Finally, the fourth parameter is the internal power supply resistance ($\:{R}_{S}$), varied from 0 Ω to 300 Ω in increments of 10 Ω, again yielding a total of 31 points. As observing parameters, the three terminal currents, as well as the emitter-base and base-collector voltages, were extracted in the dataset.

**Table 2:** Different patterns studied in this work.

Pattern	Pattern	Faulty parameter description	Parameter variation
0	Normal Pattern	-	-
1.1	Fault Pattern	Temperature ($\:T)$	25 °C:100 °C
1.2	Fault Pattern	Collector Resistor ($\:{R}_{C}$)	4.5 kΩ:4.8 kΩ
1.3	Fault Pattern	Emitter Resistor ($\:{R}_{E}$)	3.1 kΩ:3.4 kΩ
1.4	Fault Pattern	Power Supply Internal Resistor ($\:{R}_{S}$)	0 Ω:300 Ω
2.1	Fault Pattern	Temperature (T), Collector Resistor ($\:{R}_{C}$)	25 °C:100 °C, 4.5 kΩ:4.8 kΩ
2.2	Fault Pattern	Temperature (T), Emitter Resistor ($\:{R}_{E}$)	25 °C:100 °C, 3.1 kΩ: 3.4 kΩ
2.3	Fault Pattern	Temperature (T), Power Supply Internal Resistor ($\:{R}_{S}$)	25 °C: 100 °C, 0 Ω: 300 Ω
2.4	Fault Pattern	Collector Resistor ($\:{R}_{C}$), Emitter Resistor ($\:{R}_{E}$)	4.5 kΩ: 4.8 kΩ, 3.1 kΩ: 3.4 kΩ
2.5	Fault Pattern	Collector Resistor ($\:{R}_{C}$), Power Supply Internal Resistor ($\:{R}_{S}$)	4.5 kΩ: 4.8 kΩ, 0 Ω: 300 Ω
2.6	Fault Pattern	Emitter Resistor ($\:{R}_{E}$), Power Supply Internal Resistor ($\:{R}_{S}$)	3.1 kΩ: 3.4 kΩ, 0 Ω: 300 Ω
3.1	Fault Pattern	Temperature (T), Collector Resistor ($\:{R}_{C}$), Emitter Resistor ($\:{R}_{E}$)	25 °C: 100 °C, 4.5 kΩ: 4.8 kΩ, 3.1 kΩ: 3.4 kΩ
3.2	Fault Pattern	Temperature (T), Collector Resistor ($\:{R}_{C}$), Power Supply Internal Resistor ($\:{R}_{S}$)	25 °C: 100 °C, 4.5 kΩ: 4.8 kΩ, 0 Ω: 300 Ω
3.3	Fault Pattern	Temperature (T), Emitter Resistor ($\:{R}_{E}$), Power Supply Internal Resistor ($\:{R}_{S}$)	25 °C: 100 °C, 3.1 kΩ: 3.4 kΩ, 0 Ω: 300 Ω
4.0	Fault Pattern	Temperature (T), Collector Resistor ($\:{R}_{C}$), Emitter Resistor ($\:{R}_{E}$), Power Supply Internal Resistor ($\:{R}_{S}$)	25 °C: 100 °C, 4.5 kΩ: 4.8 kΩ, 0 Ω: 300 Ω, 0 Ω: 300 Ω

**Fig. 2:**
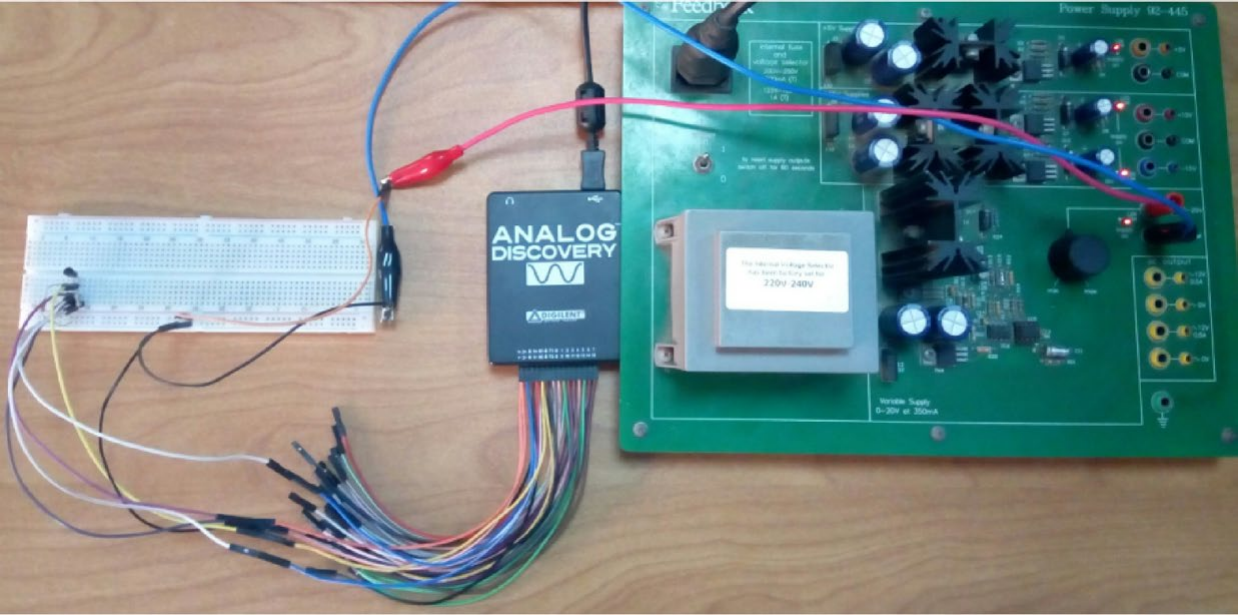
Experimental setup used to collect voltages and currents through the analog discovery acquisition system.

To address the uncertainties associated with real measurements, leading to a well-trained realistic model, we adopted experimental data gathering through our setup in Fig. 2. The 2N3904 NPN transistor from ON Semiconductor was used for the CE circuit. Moreover, the Analog Discovery 3 multi-function test and measurement device was used as a variable power supply to offer the base voltage biasing and the BJT circuit’s supply voltage for the different patterns. To extract the experimental measurements data of the circuit, Analog Discovery equipment was considered the closest ideal power source to represent the normal pattern since it has features of accuracy, stability, and low internal resistance compared to traditional power supplies. Herein, the three terminal DC voltages across the base, emitter and collector were captured, imported to csv files, and utilized for current calculations, knowing the resistance values. Moreover, the RIDEN RD6018W Complete Set was used as a traditional variable DC power supply, which can ideally represent the fluctuations of the power supply’s internal resistance by taking different readings for the fault pattern. A heat gun was applied to the circuit, causing the temperature of its components to rise by more than 25 °C. To address the variation in the collector resistance, 20 fixed carbon resistors with a typical value of 4.7 kΩ with ± 5% tolerance were used alternatively for the collector resistor; these resistors are equivalent to the parameter sweeping in the simulation tool since each resistor practically has a unique value that differs among the other resistors according to the tolerance error. For emitter resistance, 20 fixed carbon resistors with a typical value of 3.3 kΩ with ± 5% tolerance were used alternatively for the emitter resistor, following the same approach in collector resistance. Under these patterns, the three terminals’ currents and voltages were measured. Different readings were recorded for each pattern of both normal and faulty patterns and then inserted into the dataset to train the machine learning model. Therefore, a hybrid dataset model was established to be trained by the machine learning algorithm.

## Machine learning algorithms

Numerous factors must be considered before selecting a machine-learning algorithm for analog fault detection in the BJT circuit, including accuracy, interpretability, training speed, preprocessing requirements, and scalability. Out of the various algorithms considered in this work, Random Forests (RF)^17,18,50–53^, K-Nearest Neighbors (KNN)^54^, and Support Vector Machines (SVM)^55^ were selected. Choosing Random Forest among these algorithms mainly due to a targeted performance assessment based on our experimental results, competitions relevant to the performance measures, and engineering constraints imposed by the real-time fault diagnosing applications^17,18,50–53^. This ensemble learning technique builds multiple decision trees in the training phase. Every tree in the forest offers a vote for a class, and the model class prediction is the class that received the most votes. Random Forest aims to avoid overfitting by injecting randomization in the selection of training samples (bagging) and in the choice of features that are considered to split a node in each tree. This double randomization yields a robust model, has adequate generalization, and can capture varying fault conditions. Random Forest does not suffer from noise as single decision trees do but rather combines multiple weak learners to form a single strong classifier.

K-Nearest Neighbors is rank-calibrated remnant learning that does not suggest an explicit model during training. Namely, it just stores all training samples and performs the classification when an instance under test is presented by searching for the ‘k’ closest data points to it^54^. It may be simple to understand and apply, but it can be slow at any time of prediction, plus it is sensitive to irrelevant features and data distributions. The model requires computing the distance between the input and each stored sample at runtime, so the prediction time grows linearly with the number of samples, rendering the KNN unsuitable for real-time applications. On the contrary, Support Vector Machine constructs an optimal hyperplane in the multi-dimensional space, maximizing the margin between the two classes. SVC rules non-linear classification with the help of kernel functions, which map the data to a higher-dimensional space where it becomes linearly separable. In practice, SVMs are well-reputed as classifiers, especially for limited and high-dimensional datasets; however, they are computationally intensive and slow to train, exerting a high memory load when running on non-linear kernels. Furthermore, their sensitivity to the tuning of hyperparameters and scaling renders them less flexible for speedy prototyping or deploying on platforms with scant resource availability^55^.

To design an intelligent system capable of identifying failures in analog circuits made from BJTs, we adopted an approach using supervised machine learning with Random Forest classifiers for this work. The Random Forest was chosen based on a comprehensive analysis considering the key evaluating parameters used to assess ML algorithms, as detailed in Table 3. A dataset was generated as described in Sect. 3. It includes current and voltage measurements obtained under various conditions representing normal behavior to faults induced by thermal stresses and variations of circuit parameters, in combination as illustrated in Table 2. The objective of the machine learning phase is mainly to establish a model capable of consistently detecting whether a BJT circuit operates correctly or fails, based exclusively on its electrical signals, as well as identifying the fault(s). All computational processes were conducted on a cloud computational platform (Rescale: High Performance Computing) of 2x Xeon Gold 6240 2.6 GHz processor, with a total of 36 cores and 24 MB Cache, each with 32 GB RAM, supported by 2 × 480 GB SSD HD, where the computational time for all runs was in milliseconds.

**Table 3:** The evaluation parameters associated with various machine learning algorithms.

ML model	Accuracy	Precision	recall	F1-Score
Decision Tree	91.2%	0.91	0.90	0.91
Elastic Net	66.8%	0.78	0.65	0.64
Gradient Boosting	56.7%	0.58	0.56	0.59
KNN	96.5%	0.84	0.83	0.82
Lasso	-	-	-	-
Linear Regression	-	-	-	-
Ridge Prediction	93.1%	0.92	0.93	0.91
SVM	97.4%	0.77	0.68	0.63
XG-Boost Prediction	93.1%	0.69	0.64	0.55
Random Forest	98.7%	0.95	0.95	0.94

## Results and discussion

This section presents all the results extracted from this study, including both the theoretical results and the outputs of the machine learning classification model. A comprehensive analysis is provided to bridge the theoretical results with the AI findings.

### Machine learning model results

The fault-detection model created for the BJT circuit is thoroughly evaluated in this section. The findings provide insight into the model’s performance on both real hardware data and created datasets. Among these are metrics for classification accuracy, precision, recall, and F1-score that can be visually evaluated using tools such as feature significance plots, learning curves, and confusion matrices. Performance results are further examined by dataset source (hardware versus simulation), fault type, and class behavior. A significant portion of the evaluation now includes the very noises, disturbances, and real-world situations that the model had to deal with. The resulting insights confirm the robustness and dependability of the suggested AI-based diagnostic solution. The dataset used for training and evaluating the model’s performance consisted of 640,000 instances, each corresponding to a unique condition of a transistor. The training was made using six features: base voltage (V_B_), collector voltage (V_C_), emitter voltage (V_E_), base current (I_B_), collector current (I_C_), and emitter current (I_E_). Each row of the data was labeled with a class based on the classification in Table 2, depicting either normal operation or a specific fault type. The model was implemented using Random Forest classifiers. The training was conducted on 80% of the dataset, while the remaining 20% served for evaluation. This single split was implemented using the train_test_split function in scikit-learn, which confirms the randomness and balanced state of classes in both sets.

**Fig. 3:**
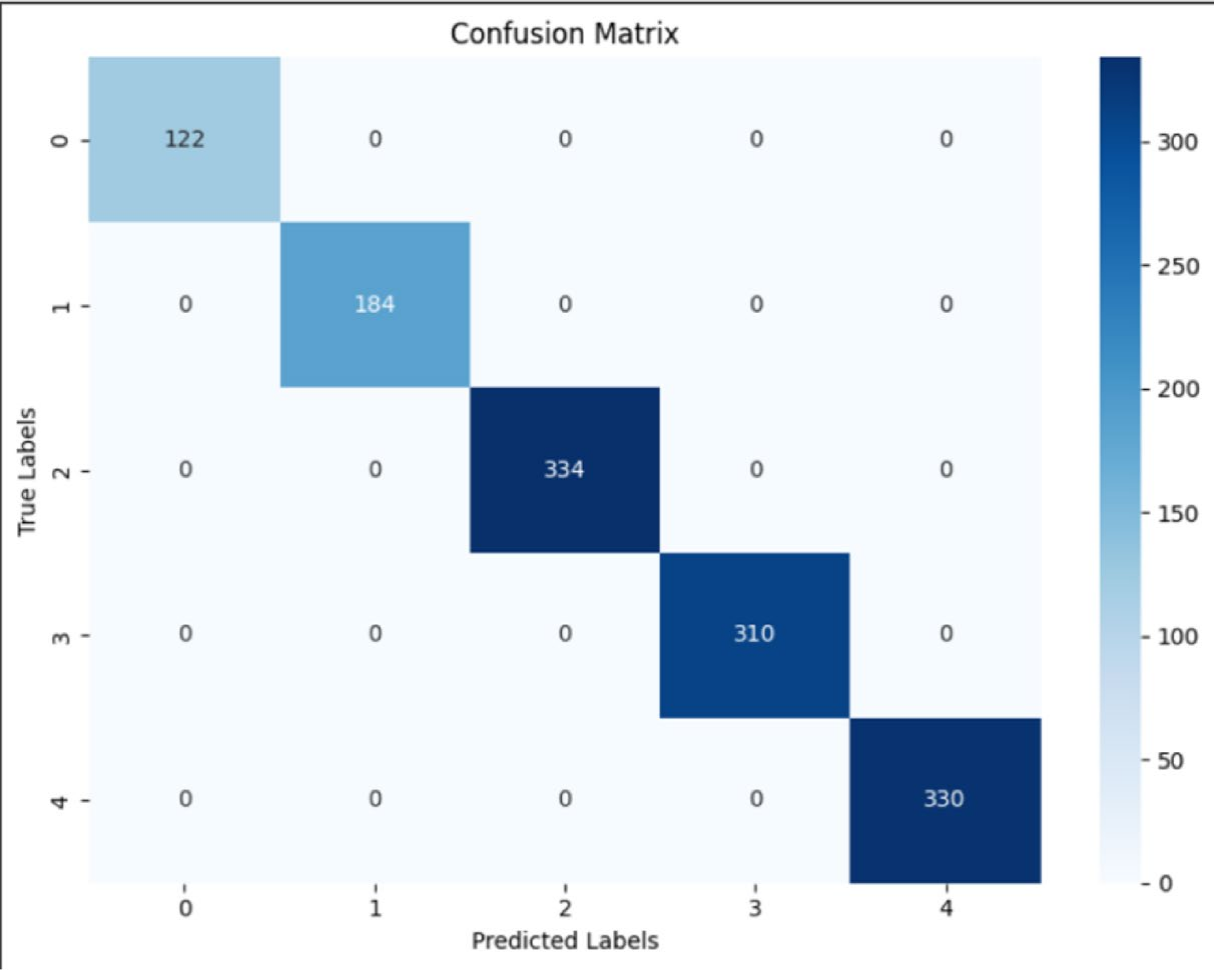
The confusion matrix for the random forest as a classification model for fault detection.

The overall performance proved to be extraordinary; the model attained 98.7% scores across all established metrics: accuracy, precision, recall, and F1 measure. Every sample tested was classified accurately. Such high precision is rare, especially in the multi-class fault-detection type. To back up the metrics regarding the confusion matrix. The Confusion Matrix showed a perfect diagonal, indicating that all predicted labels were classified correctly as actual. No false-positive observations nor false negatives were made. Thus, all the normal samples (1220 samples under class 0) were predicted correctly as normal. Similarly, all samples of thermal fault only were classified accurately (1840 samples under class 1.1), R_C_ faults (3340 samples under class 1.2), R_E_ faults (3100 samples under class 1.3), and R_B_ faults (3300 samples under class 1.4) with no errors. Thus, this matrix serves as important validation, providing evidence that the model achieved high accuracy, not in a skewed manner, but consistently across all classes. The Confusion Matrix shown in Fig. 3 provides more insights than just correctness. It visualizes important information corresponding to any main classes that confuse each other; this is a relevant notion in fault detection. For example, it would mean that overlapping behavior should be considered a thermal fault, often confused with a single fault. Each fault type produces its pattern in the electrical features, which the model learned to separate acceptably. This means that the faults themselves are distinguishable through their effects on the transistor’s voltages and currents, a key insight in developing reliable detection systems.

**Fig. 4:**
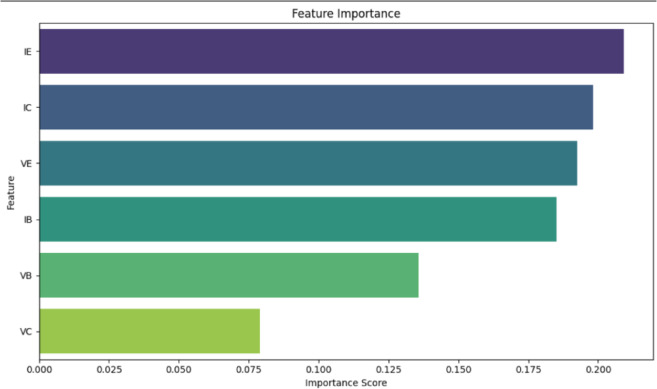
The feature importance plot for the random forest as a classification model for fault detection.

Alongside performance metrics, it is also essential to understand what exactly the model is learning. It is the feature importance plot shown in Fig. 4 that gives this insight: The graph ranked the inputs based on their contribution to the model’s decision-making process. The most important features appeared to be the emitter current I_E_, followed closely by the collector current I_C_ and the emitter voltage V_E_. Together, they accounted for most classification decisions. This result agrees with the physical behavior of the BJT. The emitter current is the summation of collector and base currents, revealing how the transistor is biased. A lot was also said concerning collector current. Since I_C_ is closely tied to the amplification behavior of the transistor, any fault that disturbs the I_C,_ such as a shift in R_s_ or R_E_, will have a strong classification effect. The voltage V_E_ came next since it changes quickly with R_E_ variation and thermal stress. The next most important features were I_B_ and V_B,_ which relate to the input characteristics of the transistor. I_B_ is an important measure of control signal strength and base biasing. V_B_ stands for the voltage at the base terminal, indicating how the transistor is turned on. Finally, V_C_ (collector voltage) turned out to be the least significant. The V_C_ effect still lingers in the model but shows less variation across fault types and remains stable, thereby reducing its discriminative power. These findings support the internal logic of the model, which learns current-driven behavior rather than arbitrary voltages and finds a reasonable correlation with known circuit theory.

**Fig. 5:**
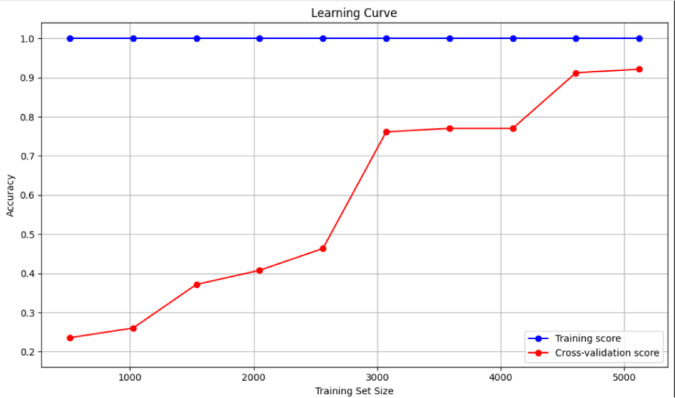
The learning curve for the random forest as a classification model for fault detection.

Another aspect of model evaluation is its learning behavior in terms of dataset size. The learning curve depicted in Fig. 5 displays such information. The blue line indicates training accuracy on the training set; the red line indicates cross-validation accuracy (the performance of the model on unseen data). The training score was 98.7% across all samples, implying that the Random Forest was capable of fully fitting the training set. However, there appears to be a little counterargument regarding the suspected overfitting, as the cross-validation line goes elsewhere. The square cross-validation score for small training sizes (e.g., 5000 to 10000 samples) would indicate that the model has not yet seen enough examples to generalize. The red line has been steadily rising since then, with the increase in data. The test accuracy exceeded 90% when the number of training samples reached approximately 30,000 to 40,000. This steady increase and subsequent convergence confirm that there is enough data, and if the model is trained correctly, it generalizes well. More interestingly, it shows no signs of high variance-the gap between training and test accuracy closed with more data, indicating that the model is not overfitting but rather learning clean decision boundaries. Therefore, these findings indicate that training was effective and that the model should be capable of producing robust results when deployed in the real world.

To validate the effectiveness of the Random Forest against other ML models investigated in this paper, accuracy comparisons from our experiment strengthened the selection of the Random Forest. RF obtained an accuracy of 98.7% through simulation, hardware, and hybrid data sets, see Table 3. KNN achieved an accuracy of approximately 96.5%, whereas SVM performed slightly better at just over 97.4%. The margin decreases considerably since, in cases where an accuracy drop of 1% could mean missing a fault or raising an alarm in critical safety environments such as fault detection in electronics, such average differences become irrelevant. Moreover, Random Forest achieves this accuracy without requiring extensive hyperparameter tuning or preprocessing, significantly reducing the burden on system developers. Over and above, the other evaluating parameters, including the precision, recall, and F1-score for Random Forest, outperform other ML algorithms, cf. Table 3.

In summary, the analysis confirms the Random Forest model’s mathematical classification, complemented by its physical behavioral concordance with the BJT in fault conditions. It learns the most relevant electrical signals, generalizes well across hardware and simulation, and maintains performance in all fault categories. Its structure facilitates fast execution, easy interpretation, and deployment in real-time embedded platforms. With its sensitivity for the high-fault diagnosis of these analog transistor systems. To assess the generalizability of the Random Forest model beyond self-generated circuit data, the external dataset prepared by some researchers working on analog circuit fault detection was classified. The model had already demonstrated 98.7% accuracy on the training and testing sets internally prepared, involving thermal and component variation faults. Investigation of this aspect aimed to examine whether the model could adapt well to new fault signatures collected from other circuit configurations and data sources. The model implemented using the Random Forest algorithm on this new dataset achieved an accuracy of 90%. This depicts high adaptability and reliability, but the performance is slightly lower than that achieved on the original dataset. The 90% accuracy on undecide, and independently generated data, attests that the model captures the important behavioral patterns of circuit faults. The external data are likely to contain other biasing setups, value ranges, or noise levels that make the task more difficult. The model still succeeded in the differentiation of fault versus non-fault conditions, further solidifying its strength in fault classification across diverse analog terrains. This experiment gives credence to the notion that the model is not overfit to the data within one context but can extend its prediction capacity to others, which is highly vital for real-life deployment and industrial fault diagnosis.

### Comprehensive analysis

**Fig. 6:**
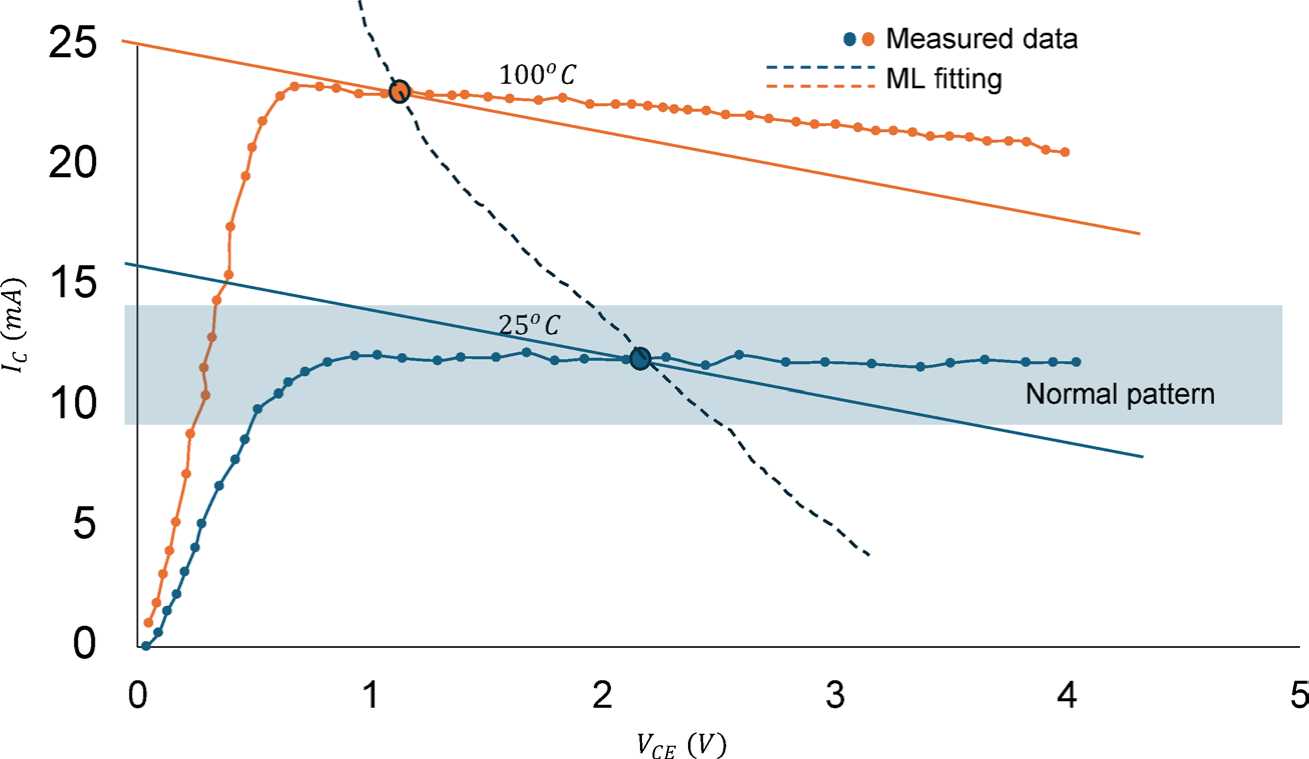
The I-V characteristics curve of a bipolar junction transistor at two different operating temperatures: 25 °C (depicted in blue) and 100 °C (depicted in orange).

**Fig. 7:**
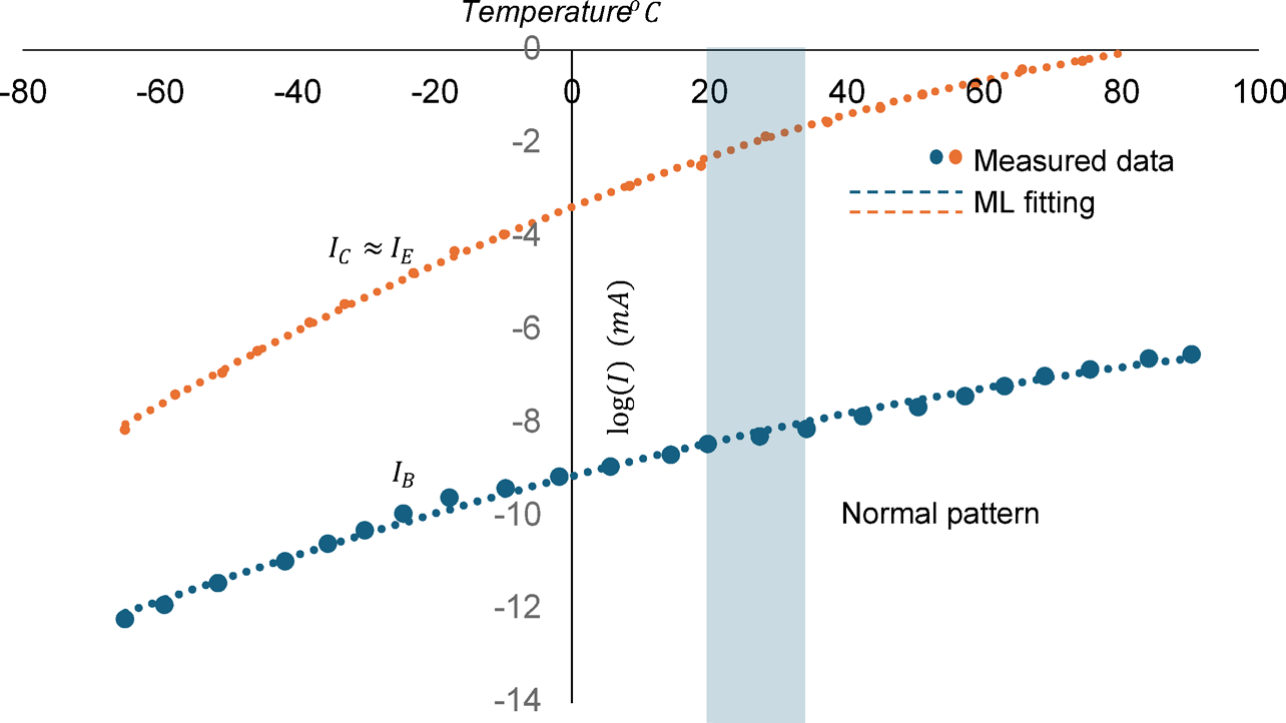
The logarithmic variation of the three terminal BJT currents concerning temperature, the theoretical (solid line), against ML outputs (dots).

Following the observations outlined in Sect. 5.1 of the AI model, we can conclude that temperature variation is the dominant factor affecting BJT operation. In this context, we will combine the outputs of the AI model with theoretical investigations to illustrate the variations in six monitoring parameters concerning temperature. Figure 6 presents the I-V characteristics curve of a bipolar junction transistor at two different operating temperatures: 25 °C (depicted in blue) and 100 °C (depicted in orange). The corresponding load line intersects with the characteristic curve at a defined operating point, demonstrating the steady-state operation of the BJT under these conditions. In contrast, the 100 °C curve indicates a higher collector current, reaching approximately 25 mA, with the transition to saturation occurring at a lower voltage compared to the 25 °C scenario. The results align with the machine learning model, highlighting the significant impact of temperature on both the BJT currents and the emitter voltage, as shown in Fig. 4. Similarly, the results in Fig. 7 indicate the logarithmic variation of the three terminal currents concerning temperature, clearly demonstrating the dominant effect of the emitter current. The theoretical results align perfectly with the ML data, exhibiting a root-mean square error (RMSE) of less than 2.2%. In both Figs. 6 and 7, the normal operating patterns are defined, indicating the temperature limits at which the circuit operating points exceed normal thresholds. The same approach was applied to the other 14 conditions, enabling the model to assess system performance under 15 different fault conditions.

**Fig. 8:**
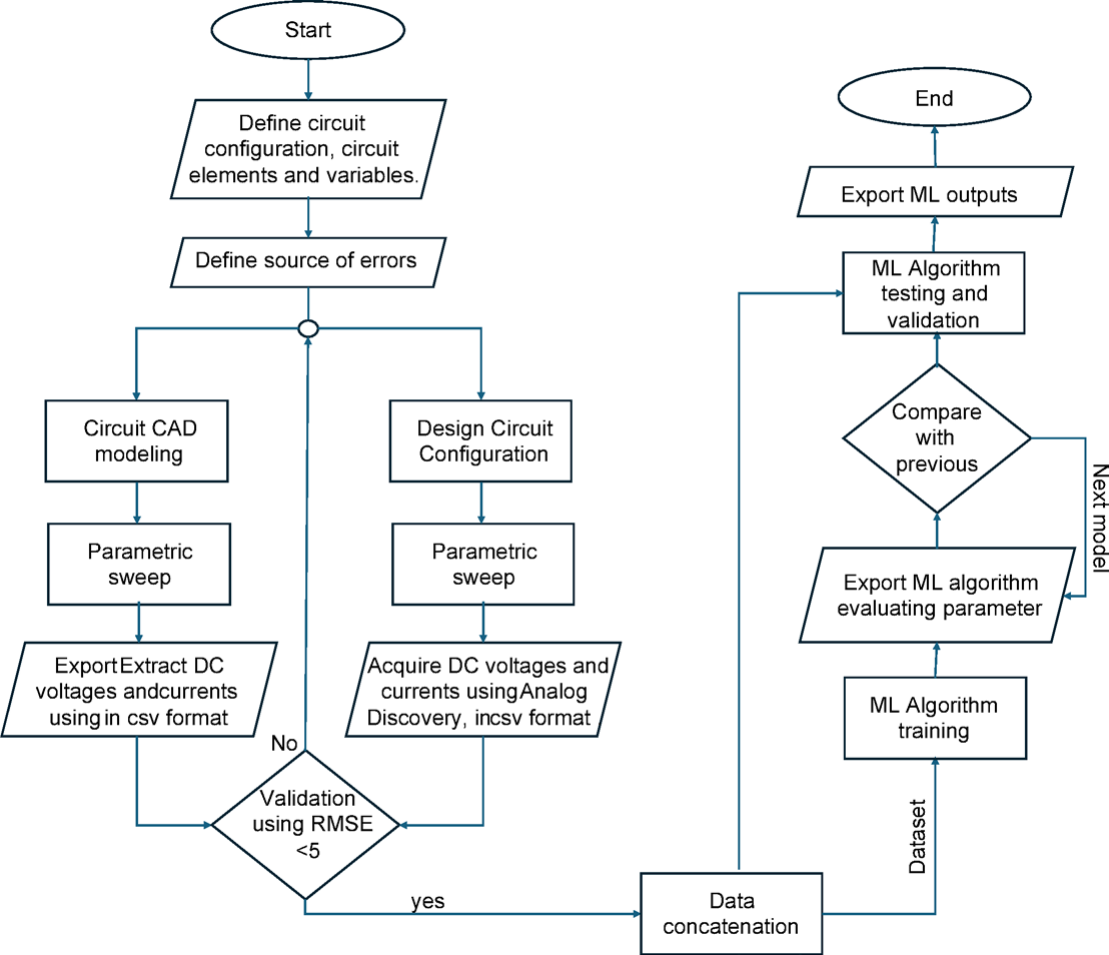
The entire AI workflow showing the hybrid dataset generation, ML training, testing, validation, and evaluation.

As the current study investigates the potential of machine learning models as a fault detection approach for BJT-based circuit configurations, it is imperative to explore the feasibility of extending this proposed methodology to encompass a broader range of BJT configurations beyond the conventional common emitter setup, including multi-stage configurations. To facilitate this exploration, we introduce a generic workflow depicted in Fig. 8. This workflow illustrates the systematic procedure for employing machine learning, commencing with the hybrid dataset collection, which integrates both simulation data and experimentally acquired data. Subsequently, we validate the CAD simulations through experimental pilot tests, followed by the concatenation of the datasets. Once the data is prepared, it is input into the machine learning algorithm for training. To assess the efficacy of the machine learning model, three evaluation parameters, as outlined in Table 3, are employed. Moreover, to ensure the robustness of the proposed methodology in exploring various datasets, output from different BJT-based configurations, we reapply our approach on three other configurations as listed in Table 4. Herein, the data in Table 4 validate the computability of the model to be extended to other circuit configurations. Still, to test our model acceptability to different dataset natures, we adopted three online available datasets^56–58^ representing fault detection in analog circuits to fit our approach. Corresponding results are displayed in Table 4. Although the selected datasets are related to AC circuit operation, our model could show acceptable performance. This comprehensive approach not only enhances the robustness of the fault detection process but also paves the way for future research into diverse BJT configurations. Future extensions might include, but not be limited to, enabling trainset circuit analysis, with an appropriate analytical approach, and experimental data acquisition in the transient phase operation. In addition, future work can evolve AC operation for amplifiers with both BJT and MOSFET fault detection.

**Table 4:** The evaluation parameters associated with various circuit configurations explored under random forest machine learning algorithms.

Configuration	Accuracy	Precision	Recall	F1-Score
Common-emitter	98.7%	0.95	0.95	0.94
Common-base	99.1%	0.98	0.99	0.98
Common-collector	98.4%	0.99	0.98	0.97
Multi-stage amplifier	98.3%	0.95	0.93	0.93
Dataset in^56^	96.3%	0.96	0.96	0.95
Dataset in^57^	94.7%	0.92	0.94	0.95
Dataset in^58^	91.5%	0.91	0.90	0.91

## Conclusion

In conclusion, this study has successfully developed and evaluated an AI-based fault diagnosis system specifically designed for analog circuits, with a particular focus on bipolar junction transistor (BJT) configurations. The findings indicate that temperature variation is the predominant factor influencing BJT operation, as evidenced by the significant differences in I-V characteristics at varying temperatures. The analysis revealed that at 100 °C, the collector current increased to approximately 25 mA, with saturation occurring at a lower voltage compared to the 25 °C scenario, confirming the machine learning model’s predictions. The theoretical results demonstrated a strong correlation with the machine learning data, achieving a root-mean square error (RMSE) of less than 2.2%. Furthermore, the comprehensive evaluation of multiple BJT configurations, including common-emitter, common-base, common-collector, and multi-stage amplifier setups, showcased the model’s high accuracy (ranging from 98.3% to 99.1%) in distinguishing between normal and faulty circuit behaviors. This study not only validates the effectiveness and robustness of the proposed machine learning approach but also highlights its potential for broader applications in analog circuit diagnostics. Future work will focus on expanding the model’s generalizability to encompass a wider range of analog circuits, enhancing its adaptability to different topologies and signal behaviors. Additionally, the development of a multi-class classification system to categorize fault severity levels will increase the model’s utility in predictive maintenance, enabling early detection of component degradation. Ultimately, the goal is to create a self-updating model that learns from new data, ensuring long-term reliability and flexibility in dynamic operational environments.

## Data Availability

The data supporting this study’s findings are available from the corresponding author upon reasonable request.

## List of symbols

$\:B$: The base intrinsic terminal of the BJT transistor considering the parasitic resistance effect
$\:B{\prime\:}$: The base intrinsic terminal of the BJT transistor, without considering the parasitic resistance effect
$\:C$: The collector intrinsic terminal of the BJT transistor, considering the parasitic resistance effect
$\:C{\prime\:}$: The collector intrinsic terminal of the BJT transistor, without considering the parasitic resistance effect
$\:{C}_{B{\prime\:}C{\prime\:}}$: The depletion capacitance of the base-collector junction is characterized as the combined effect of the space-charge capacitance and the diffusion capacitance components (nF)
$\:{C}_{B{\prime\:}E{\prime\:}}$: The depletion capacitance of the base-emitter junction is characterized as the combined effect of the space-charge capacitance and the diffusion capacitance components (nF)
$\:{C}_{JC}$: The zero-bias capacitance of the base-collector junction (nF)
$\:{C}_{JE}$: The zero-bias capacitance of the base-emitter junction (nF)
$\:E$: The emitter intrinsic terminals of the BJT transistor, considering the parasitic resistance effect
$\:E{\prime\:}$: The emitter intrinsic terminals of the BJT transistor, without considering the parasitic resistance effect
$\:{E}_{G}$: The bandgap energy (eA)
$\:{I}_{B}$: The base current (mA)
$\:{I}_{B{\prime\:}C{\prime\:}}$: The base-collector current (mA)
$\:{I}_{BCrec}$: The recombination current through the base-collector junction (mA)
$\:{I}_{B{\prime\:}E{\prime\:}}$: The base-emitter current (mA)
$\:{I}_{BErec}$: The recombination current in the base-emitter junction (mA)
$\:{I}_{C}$: The collector current (mA)
$\:{I}_{E}$: The emitter current (mA)
$\:{I}_{F}$: The ideal forward diffusion current (mA)
$\:{I}_{KF}$: The forward knee current (mA)
$\:{I}_{KR}$: The reverse knee current (mA)
$\:{I}_{R}$: The ideal reverse diffusion current (mA)
$\:{I}_{RB}$: The base current at which the base resistance$\:{\:r}_{BB}\left({I}_{B}\right)$ decreases to the mean value between $\:{r{\prime\:}}_{B}$ and $\:{r}_{BM}$ (mA)
$\:{I}_{S}$: The saturation current (pA)
$\:{I}_{SC}$: The leakage current of the collector (mA)
$\:{I}_{SE}$: The leakage current of the emitter (mA)
$\:k$: Boltzmann constant (eV/K)
$\:{M}_{JC}$: The base-collector junction exponential factor (1)
$\:{M}_{JE}$: The base-emitter junction exponential factor (1)
$\:{N}_{C}$: The collector current emission coefficient (1)
$\:{N}_{E}$: The emitter current emission coefficient (1)
$\:{N}_{F}$: The forward current emission coefficient (1)
$\:{N}_{qb}$: The base charge formula (1)
$\:{N}_{R}$: The reverse current emission coefficient (1)
$\:{n}_{i}$: The intrinsic carrier concentration (cm^-3^)
$\:q$: The unit charge (C)
$\:{\text{q}}_{1\text{s}}$: Models the base-width modulation (1)
$\:{\text{q}}_{2\text{s}}$: The high-level injection effect (1)
$\:{r}_{BB}\left({I}_{B}\right)$: The parasitic intrinsic base resistance as a function of base current ($\Omega$)
$\:{r}_{BM}$: The minimum base resistance at high base current ($\Omega$)
$\:{{r}^{{\prime\:}}}_{B}$: The parasitic intrinsic base resistance at zero bias base ($\Omega$ $\Omega$)
$\:{R}_{C}$: The extrinsic collector resistance ($\Omega$)
$\:{{r}^{{\prime\:}}}_{C}$: The parasitic intrinsic collector resistance ($\Omega$)
$\:{R}_{E}$: The extrinsic emitter resistance ($\Omega$)
$\:{{r}^{{\prime\:}}}_{E}$: The parasitic intrinsic emitter resistance ($\Omega$)
$\:{R}_{S}$: The base bias source resistance ($\Omega$)
$\:T$: The operating temperature ($\Omega$)
$\:{T}_{0}$: The reference temperature ($\Omega$)
$\:{V}_{AF}$: The forward early voltage (V)
$\:{V}_{AR}$: The reverse early voltage (V)
$\:{V}_{BC}$: The base-collector voltage (V)
$\:{V}_{\text{B'C'}}$: The intrinsic base-collector junction voltage (V)
$\:{V}_{BE}$: The base-emitter voltage (V)
$\:{V}_{\text{B'E'}}$: The intrinsic base-emitter junction voltage (V)
$\:{\:V}_{th}$: The thermal voltage (mV)
$\:XTB$: The temperature exponent for the effect of $\:{I}_{S}$ reverse-beta temperature exponent (1)
$\:XTI$: The temperature exponent for the effect of $\:{I}_{S}$, forward-beta temperature exponent (1)
$\:{\beta\:}_{F}$: The ideal forward maximum current gain (1)
$\:{\beta\:}_{F}\left({T}_{0}\right)$: The forward common-emitter current gain at the standard reference temperature (1)
$\:{\beta\:}_{R}$: The ideal reverse maximum current gain (1)
$\:{\beta\:}_{R}\left({T}_{0}\right)$: The reverse common-emitter current gain at the standard reference temperature (1)
